# Current Knowledge on Chemosensory-Related Candidate Molecules Potentially Involved in Tick Olfaction via Haller’s Organ

**DOI:** 10.3390/insects14030294

**Published:** 2023-03-18

**Authors:** Mebrahtu Berhe Gebremedhin, Zhengmao Xu, Ceyan Kuang, Nigus Abebe Shumuye, Jie Cao, Yongzhi Zhou, Houshuang Zhang, Jinlin Zhou

**Affiliations:** 1 Key Laboratory of Animal Parasitology of Ministry of Agriculture, Shanghai Veterinary Research Institute, Chinese Academy of Agricultural Sciences, Shanghai 200241, China; 2 State Key Laboratory of Veterinary Etiological Biology, National Animal Echinococcosis Para-Reference Laboratory, Key Laboratory of Veterinary Parasitology of Gansu Province, Lanzhou Veterinary Research Institute, Chinese Academy of Agricultural Sciences, Lanzhou 730046, China

**Keywords:** candidate binding proteins, candidate chemoreceptors, Haller’s organ, limitations, tick

## Abstract

**Simple Summary:**

Chemosensation is thought to be the primary sensory mechanism used by ticks to locate their host and partners, as they appear to lack functional eyes or hearing organs, in which their unique olfactory structure, namely Haller’s organ, is considered to play a significant role, although little is known about its molecular biology. This review comprehended and discussed the existing molecular information on candidate chemoreceptors and potential binding proteins preferentially expressed in tick foreleg enriched Haller’s organ, backed by pertinent bioinformatic data. Additionally, tick olfactory-related structural, behavioral, and neurological information was also given, and its limits were highlighted. Despite their expression in the forelegs of ticks, independent functional experiments should confirm the olfactory role of these chemosensory-related candidate molecules.

**Abstract:**

Ticks are obligatory hematophagous ectoparasites and vectors of many animal and human pathogens. Chemosensation plays a significant role in tick communication with their environment, including seeking out blood meal hosts. Studies on the structure and function of Haller’s organ and its components have improved our understanding regarding tick olfaction and its chemical ecology. Compared with the knowledge on insect olfaction, less is known about the molecular basis of olfaction in ticks. This review focused on the chemosensory-related candidate molecules likely involved in tick olfaction. Members of the ionotropic receptor family and a new class of odorant-binding proteins are now known to be involved in tick olfaction, which appear to differ from that of insects. These candidate molecules are more closely related to those of mites and spiders than to other arthropods. The amino acid sequences of candidate niemann–pick type C2 and microplusin-like proteins in ticks exhibit features indicating their potential role as binding proteins. In the future, more comprehensive pertinent research considering the existing shortcomings will be required to fully understand the molecular basis of tick olfactory chemoreception. This information may contribute to the development of new molecular-based control mechanisms to reduce tick populations and related disease transmission.

## 1. Introduction

Ticks are ectoparasites and worldwide vectors of animal and human pathogens [1,2]. Ecological and geographical variables influence both the genetic makeup and pathogens transmitted by ticks [2], and global warming may complicate the problems of ticks and tick-borne diseases (TBDs) [3]. Many ticks feed on domestic animals and cause significant economic losses. *Rhipicephalus microplus* is responsible for a quarter of all veterinary ectoparasite infections worldwide [4,5]. TBDs cause substantial public health concerns. In addition, the unknown health repercussions and lack of control methods for newly emerging TBDs generate human anxiety [6]. The geographic range of zoonotic TBDs is expanding and infection rates could increase in the future [7].

Ticks must be able to recognize suitable hosts to obtain blood meals that are needed for survival and reproduction. Host feeding allows pathogens to be transmitted to the host [8,9]. Vision seems less important than odor in ticks [10], and they do not appear to have hearing organs because they lack the transient receptor potential vanilloid (TRPV) mechano-gated channel that transduces sound in both insects and vertebrates [11]. Hence, controlling tick and tick-borne diseases poses difficulties [12]. Olfactory chemosensation in ticks appears to be the sensory mechanism that contributes most to host-seeking [13]. The integration of chemoreceptors with other related receptor signals is also thought to be important [14]. Interference in tick olfactory chemoreception could have a direct impact on the tick’s ability to recognize hosts and potentially impede tick biting.

Most tick olfaction-related studies have focused on the structure and function of Haller’s organ [14], which was assumed to contain the olfactory receptor molecules. The olfactory system, particularly Haller’s organ, plays a significant role in tick responses to attractant chemicals [14]. Haller’s organ may also be a potential target for the development of novel tick repellents [15,16]. Understanding how ticks interact with their surroundings is essential for developing new control mechanisms [14]. However, inadequate molecular knowledge is an obstacle to the use of olfaction for tick control [17].

Most arthropod olfaction research has focused on insects, and most of the proteins involved have been identified in insects. However, the tick molecular chemoreception is a recent topic of research interest. Haller’s organ-targeted transcriptome and proteome analysis have identified several candidate chemosensory-related molecules involved in tick olfaction (Table 1) since the genome of *Ixodes scapularis* was sequenced [18]. This review compiled the olfaction-related molecular information from ticks, compared these data with data from insects, and highlighted possible study limitations. The information may provide direction to researchers regarding advanced studies of tick olfaction.

## 2. Tick Olfaction and Haller’s Organ

### 2.1. Haller’s Organ: Olfactory Role and Its Structural Divergence

Most olfactory-related research in ticks has focused on the structure and function of Haller’s organ [14]. Haller’s organ is a unique sensory structure located on the fore tarsus of the first pair of tick legs. Thereby, in this review paper, the chemosensory-related candidate molecules differentially expressed in the fore-tarsal appendage of ticks are mentioned as that of Haller’s organ. This organ has anterior pit and posterior capsule regions, and contains several chemosensory sensilla [16]. Among these chemosensory sensilla, olfactory sensilla, a single-walled multi-pore sensilla, have been reported to primarily reside in Haller’s organ [19,20]. These sensilla mainly detect odorant molecules [17].

Several studies have confirmed the olfactory role of Haller’s organ in ticks [13,14,18,21], which guides them in locating their hosts [17]. In addition to their response to attractants [14], a transcriptome analysis of *Dermacentor variabilis* has provided molecular evidence revealing the fundamental role of Haller’s organ in spatial DEET repellence [22]. Therefore, the major contribution of Haller’s organ in tick olfactory chemoreception, possibly in the host-seeking role of ambushing ticks, seems evident. Haller’s organ may therefore be a target for olfactory-based tick control studies.

Scanning electron microscopy has been widely used to study the Haller’s organ structure in ticks. Considerable morphological differences were found among Haller’s organs of *I. scapularis*, *A. americanum*, and *D. variabilis* [23], and sexual dimorphism was even observed within the later tick species. This appears to contradict Leonovich [24], who concluded that Haller’s organ structure differs between genera, but not among species. The number of sensilla in the anterior pit region of Haller’s organ of *Dermacentor reticulatus* varies in postembryonic stages, in addition to displaying differences in topography, size, and surface structure [25]. The authors suggest that this may relate to life cycle, feeding, host specificity, and habitat differences. It has been suggested that the structural appearance within Haller’s organ alters the olfactory role of its specific region, particularly the posterior capsule [26]. The physical and compositional divergence of Haller’s organ may directly impact its role and reflect the tick’s ability to recognize hosts.

### 2.2. Olfactory Glomeruli and Its Neural Projection from the Haller’s Organ

The paired olfactory lobes are made up of glomeruli, situated in the ventral side of the synganglion, medial to the first pedal ganglia, and posterior to the esophagus entrance [27,28]. Recent three-dimensional (3D) reconstruction of *Ambylomma sculptum* olfactory lobes revealed a corresponding round-shaped glomeruli between 24 and 30 [28], which was half lower than some hematophagous insects [29,30], but relatively higher (16–22) than those of *Amblyomma americanum* [19], and the predatory mite, 14–21 [31]. This number was believed to be related to the amount of chemical signal repertoires they were able to perceive [32]. Additionally, a sexual variation in the number of olfactory glomeruli in ticks was noted [28], which was consistent with an earlier study of sexual dimorphism in relation to the Haller’s organ structure [23]. In the future, studies on tick odorant response, olfactory structure, and other pertinent topics should take this into account.

The olfactory glomeruli receive projections from the olfactory sensilla in the Haller’s organ [28]. They have been identified as the terminal arborizations of sensory neurons arising from these olfactory sensilla, according to neuronal tracers loaded from the organ [19,28,33]. Serotonin (5HT), a neurotransmitter that interacts with several receptors to mediate a variety of responses [34]. Serotonin-like immunoreactivity has been observed in the synganglion of ticks with a similar pattern to that of spiders. Additionally, serotoninergic neuronal processes around the olfactory glomeruli have been observed using neurobiotin backfilled from the Haller’s organ [28]. In general, the neurobiotin backfill and immunocytochemistry have revealed an olfactory lobe structure in ticks comparable with that of mites and insects, in which serotonin modulates the response to chemical signals received by the antennal lobes, suggesting that serotonin may also play a similar role in ticks [28]. However, its abundance outside of the olfactory lobes [28], which has been confirmed to bind insect-like GPCRs [35], has also been reported to be involved in tick feeding [36], indicating that serotonin in ticks has several functions. More comprehensive and pertinent cellular, molecular, and immunological experiments are needed to discover the prospective neurotransmitters and other secreted molecules underpinning the sensory neurons connected to Haller’s organ and to validate their particular function in the processing of olfactory stimuli.

Neurophysiological experiments that target the olfactory sensilla in Haller’s organ have demonstrated the sensitivity of the olfactory receptor neurons to attractant compounds. Despite the fact that the olfactory organ and the types and numbers of its sensilla are thought to be unique to acarine [14], tick olfaction may be cellularly comparable to that of insects. In fact, neural and sensory function-related information in ticks is scarce [28]. These evidences related to the anatomical basis of olfactory glomeruli and their neural projection from the Haller’s organ in ticks may improve our understanding on the mechanisms of olfactory-related sensory information processing in ticks.

### 2.3. Tick Behavioral and Chemosensory Responses to Host-Related Attractants

#### 2.3.1. Behavioral Response to Host Odor and Related Attractants

Kairomones, such as those in host breath [37], dermal pelage [38], gland secretion, and urine [39,40], can attract ticks. Among the attractant components of human and animal breath, acetone, ammonia (NH3), carbon dioxide (CO_2_), and 1-octen-3-ol significantly attract *A*. *americanum* and *D. variabilis*, while CO_2_ consistently attract the highest number of ticks [41].

Faraone et al. [42] showed that *Ixodes ricinus* was attracted to several mammalian odors, and *I. scapularis* was attracted to butyric acid. *Rhipicephalus microplus*, which exclusively parasitize bovines, were attracted to 1-octen-3-ol, which is also associated with bovine breath and odors [43]. Behavioral bioassays in the laboratory and the field have demonstrated the effectiveness of CO_2_ for collecting host-seeking ticks [44]. These data indicated the significant role of the olfactory system in tick chemical ecology and possibly host-seeking. Nevertheless, host-related attractants in ticks have received relatively less attention compared with the practical utilization of insect pheromones. Additionally, host-related attractants could have relatively wider dynamics than pheromones, which are species-specific [14]. The intended interventions targeting the receptor proteins of ticks may potentially alter their host-seeking behavior, thereby preventing the hosts from a bite and related risks.

#### 2.3.2. Neurophysiological Response to Host-Related Attractants; Using Haller’s-Organ-Based Olfactory Sensilla

Electrophysiological studies can show chemosensory cell activity, identify regions and cells involved in odor detection, and screen for attractant and repellent compounds [14]. Here, we summarize experimental findings of electrophysiological responses of neural cells to host-related odorants, targeting olfactory sensilla that are localized in Haller’s organ of ticks.

Olfactory receptor neurons (ORNs) of the multi-porous sensilla located anterior to and within the anterior pit of Haller’s organ in *Amblyomma cajennense* respond to nonanal and 1-octen-3-ol host-associated volatiles [20]. In *I. ricinus*, two distinct types of ORNs from the distal knoll of Haller’s organ were stimulated by phenol and lactone derivatives extracted from steer wool odor and were assumed to allow host perception from a distance of 10–15 m [45]. Additionally, three ORNs in the capsule region of Haller’s organ from *Ixodes scapularis* have shown high excitatory responses to phenol-rich deer gland extracts, while methyl-phenols have produced greater responses [46]. The data showed the tick’s ability to identify many volatile compounds by a single multi-pore sensillum (olfactory sensilla) located in Haller’s organ. Bohbot and Dickens [47] also found that insect olfactory receptors react to specific host-associated volatile components.

Behavioral and electrophysiological studies have revealed the significant role of the olfactory system in tick chemical ecology. The olfactory system mainly involves Haller’s organ and olfactory receptor neurons in response to odors emitted by hosts. This may help to identify new chemosensory molecules that expedite the development of an alternative molecular-based tick control mechanism.

## 3. Candidate Chemoreceptors Biasedly Expressed in Haller’s Organ

### 3.1. Ionotropic Receptors (IRs)

IRs are an olfactory version of the widely distributed ionotropic glutamate receptors (iGluR). They form ion channels and are the second-largest group of chemoreceptors in insects and other arthropods [48]. IRs are composed of an extracellular ligand-binding domain and three transmembrane domains [49] known to be involved in olfaction [50,51]. Gulia-Nuss et al. [18] sequenced the genome of *I. scapularis* and found 15 genes that likely belong to the putative chemosensory ionotropic receptor (IR) subfamily. Another large-scale genome analysis from six tick species [2] also reported multiple putative chemosensory-related genes encoding relatives of IRs, forming six tick genomes.

Josek et al. [52] discovered highly selective and divergent IR family members, with several genes exclusively expressed in the foreleg transcriptome of *I. scapularis.* They suggested that these molecules contribute to the chemosensory function of Haller’s organ. This tick foreleg transcriptome analysis extended (Ir101–Ir156) the number of IR genes obtained from the *I. scapularis* genome [18]. Among the existing tick chemosensory-related candidate chemoreceptors, members of the IR family have been reported to be potentially involved in olfaction, with relatively few gustatory receptors (GRs) and no odorant receptors (ORs). Among these IRs, Ir25a- and Ir93a-like proteins of *I. scapularis* were insect-like, highly conserved molecules reported from ticks, to date. They have shown sequence similarity with their counterparts from some insects [53,54] and crustaceans [55], characterized as olfactory/chemosensory proteins. Thus, olfactory chemoreception via the tick Haller’s organ appears to heavily rely on ionotropic receptors (IRs). However, relevant functional experiments should confirm the olfactory role of each candidate molecule.

### 3.2. Gustatory Receptors (GRs)

GRs are genes [56] that mediate the perception of diverse stimuli in insects. These stimuli are mostly gustatory and include some chemical compounds like CO_2_ [57]. They are related to the OR families and likely consist of the seven transmembrane domains of GPCR super-families. However, they may function as ligand-gated ion channels [58], possibly by combining parts of their transmembrane domains and the extra-cellular loops that connect them [59]. Despite that, the read counts for GRs were fairly lower than those for candidate IRs. A foreleg transcriptome analysis of *I. scapularis* showed that genes encoding gustatory receptors (GRs) displayed consistent foreleg bias in both male and female ticks [52]. These genes may contribute to the olfactory role of Haller’s organ, as authors have suggested. Hence, we suggest that because GRs are implicated to biasedly express in Haller’s organ of *I. scapularis*, future extensive studies need to determine their level of significance in overall tick olfactory chemoreception.

### 3.3. G-Protein Coupled Receptors (GPCRs)

GPCRs are the largest protein family in the human genome. They convert extracellular signals into important physiological outcomes [60]. GPCRs include potential target molecules for insecticide development [61,62]. In the case of ticks, GPCRs have been bioinformatically predicted from a foreleg transcriptome of *Rhipicephalus microplus* [63]; however, their level of expression was not quantified. Meanwhile, a foreleg and putative Haller’s-organ-specific transcripts were also identified from adult *D. variabilis* [16], and the authors proposed that components of a GPCR pathway are involved in tick olfaction.

However, these candidates were not found by Josek et al. [52]. They investigated the expression levels of the abovementioned candidate proteins orthologues of *I. scapularis* and reported that none of these genes showed foreleg bias in either sex. In addition, none of the tick genome or other relevant transcriptome and proteomic study findings have reported a role of GPCRs in tick olfaction. Thus, GPCRs might not be chemoreceptors in tick olfactory reception. However, their expression profile in Haller’s organ and related biological and cellular level functions need confirmation.

Which pathway mediates tick olfactory reception via Haller’s organ? Unlike that of insects, the molecular mechanisms of olfactory chemoreception in ticks is unknown. According to Carr et al. [16], who reported a GPCR pathway cascade specific to Haller’s organ, tick olfaction via Haller’s organ may use a GPCR pathway similar to nematodes and vertebrates. However, Josek et al. [52], who did not find these candidate GPCRs, rather widely proposed chemoreceptors of the IR family, and possibly the GR, which were evolutionarily linked to those of mites. They then suggested that ligand-gated ion channels mediate tick olfaction, similar to other arthropods. In addition to the absence of these candidate GPCR expressions in Haller’s organ of *I. scapularis*, all of the molecular information from tick genomes and olfactory organ-based transcriptome and proteomic evidence supports the latter hypothesis. However, the variability of candidate molecules among tick species indicates the need for targeting GPCRs, and the presence of a critical knowledge gap in understanding tick olfactory pathways.

## 4. Potential Binding Proteins Expressed in Haller’s Organ

### 4.1. Niemann–Pick Protein Type C2 (NPC2s)

NPC2s act as a semi-chemical binding and transport protein in arthropods, including insects, as recently documented [64,65,66]. Pelosi et al. [64] proposed NPC2s as a new family of binding proteins with a potential role in tick olfaction, especially Haller’s organ. This proposal was supported by olfactory organ targeted foreleg transcriptome and proteome studies from several tick species, particularly *I. ricinus*, *A. americanum*, and *I. scapularis*(Table 1). Recent tick genomic data also revealed several putative chemosensory-related genes encoding NPC2 proteins [2].

**Figure 1:**
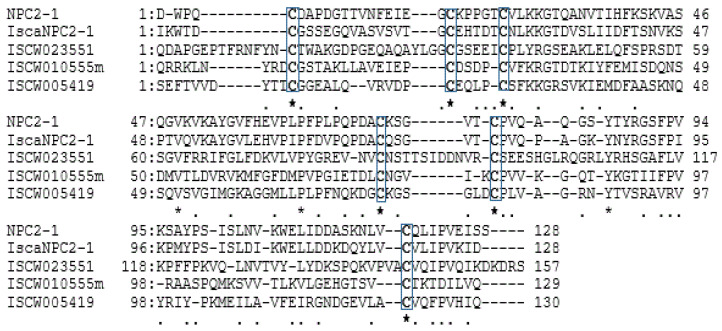
Multiple-sequence alignment analysis on candidate NPC2s likely involved in tick olfaction. Amino acid sequences have been aligned to emphasize the locations of shared cysteine in the mature proteins of the candidate NPC2s; marked with a box and asterisked. Source, NPC2-1 from *A. americanum* [67]; IscaNPC2-1 from *I. ricinus* [69], while the remaining NPC2s were from *I. scapularis* [52]. Protein sequences and related information regarding an individual gene are available in their respective publications. As mentioned above, we retained the same gene name. Multiple alignment was computed using Genetyx.6.

Several NPC2s were found to be deferentially expressed in the fore tarsus and palps of *A. americanum* [67] and *I. scapularis* [52]. Immunohistochemistry tests have demonstrated selective expression of NPC2s in specific sensilla in Haller’s organ, and were also expressed in non-olfactory organs of ticks. This feature is also shared by those of insect’s chemosensory proteins [68]. A ligand-binding assay revealed the high affinity of NPC2 in *I. ricinis* to bind with some common volatile compounds [69], and the NPC2s belong to multigene families. They also contain six conserved cysteines (Figure 1), likely to form a three disulfide bonds which may enable them stay stable in the extra cellular environment. In addition to their biased expression in Haller’s organ, the NPC2 characteristics indicated that they might act as binding proteins potentially involved in tick olfactory chemosensation. Relevant functional experiments need to confirm this.

### 4.2. Microplusin-like (ML) Proteins

**Figure 2:**
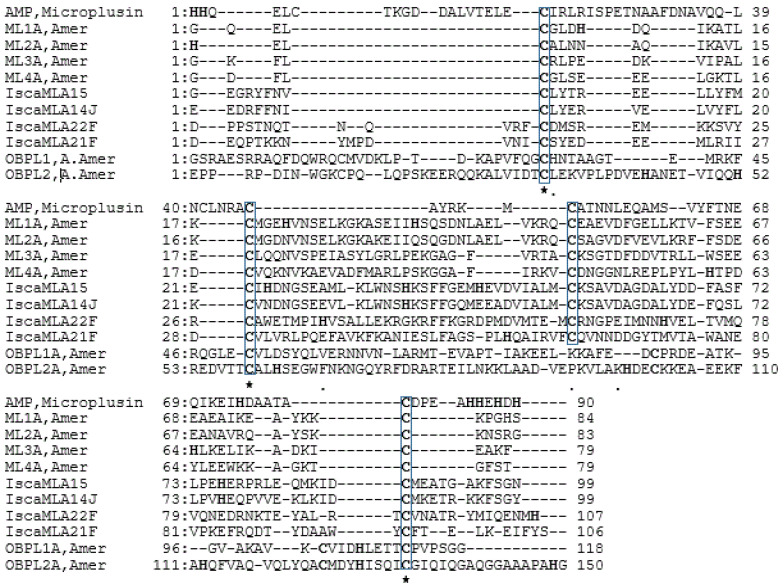
Multiple-alignment analysis on candidate ML proteins likely involved in tick olfaction. Protein sequences of candidate microplusin-like (ML) molecules were aligned to highlight the number and positions of shared cysteine and histidine residues. Conserved cysteines are boxed and asterisked, and histidines are boldly highlighted. ML protein reported from *A. americanum* [67], and members of the MLA family group from *I. scapularis* [52] are represented in this analysis; similar gene names are retained. Candidate ML proteins were also compared with the precursor antimicrobial peptide, microplusin (AMR, Microplusin) (XP_037269734.1), and OBPL-1; JZ183505 and OBPL-2; JZ172282 [67]. Multiple-alignment analysis was computed using Genetyx.6 software.

Similar to NPC2s, ML proteins have also been reported from a foreleg transcriptome analysis [52] of *I. scapularis* and a proteome analysis of *Amblyomma americanum* [67], as well as large-scale, tick genomic [2] studies. ML proteins may be involved in tick olfaction. Unlike the antimicrobial peptide microplusin, none of the tick ML proteins shared the same number and position of histidine residues (Figure 2). This is in line with the result of Renthal et al. [67], who found a similar divergence in histidine residues of ML proteins from *A. americanum*. Therefore, these ML proteins in ticks lack the copper-binding site, which is critical for their antimicrobial activity [70].

Genes encoding candidate ML proteins have been exclusively or differentially expressed in the fore tarsi or in the palps [67]. However, sometimes they are not present in either tissue [52], similar to that of candidate NPC2s. They have been modeled with an interior cavity and a possible ligand-binding site [67]. At the same time, a foreleg transcriptome analysis of *I. scapularis* [52] revealed numerous transcripts encoding eight divergent ML protein families (designated MLA-H). Their structural pattern is shared with two structurally related odorant-binding protein-like (OBPLs) proteins [67]. In addition to their exclusive and differential expression in Haller’s organ, the presence of a comparable ligand-binding site and the absence of antimicrobial basics might indicate their potential role in tick olfactory chemosensation. Relevant functional experiments need to confirm this.

### 4.3. Odorant-Binding Protein-like (OBPL) Proteins

A chemosensory appendage proteome of *A. americanum* [67] and homology modelling revealed two foreleg-biased proteins that resemble insect odorant-binding proteins. N-terminal signal sequences were predicted from their mature proteins, OBPL-1 (19 aa; 118 aa) and OBPL-2 (24 aa; 150 aa), and indicated that they were small secreted proteins that shared three of their cysteine residues (Figure 2). They appeared to possess structural similarities with ML proteins that have been reported to be involved in tick olfaction [52]. Tick OBP-like proteins have been predicted to have a comparable odorant-binding cavity and also have four cysteines with similar patterns to insect odorant-binding proteins (OBPs) [67].

Despite retaining the abovementioned features of OBPs, these candidate OBPLs lack sequence similarity with insect OBPs [67]. The authors of this study hypothesize that this might be because these OBPLs in ticks may be evolved to such a level that they cannot be detected by BLAST analysis, or that ticks may recruit a new type of binding protein, unlike that of insects. In addition, only two candidate genes encoding the OBPLs have been identified, and they do not belong to a multi-gene family. Additionally, no gene-encoding OBPs have been detected from any tick genome. However, a new class of binding proteins (NPC2s and ML) has been proposed to be involved in tick olfaction [52,67,69]. Therefore, these evidence the possible olfactory role of these insect-like OBPs in tick, which needs to be further characterized by relevant functional experiments.

### 4.4. Binding Protein Features Shared by the Candidate NPC2s and MLs Expressed in Haller’s Organ

NPC2s and ML proteins have biased expression in tick foreleg enriched Haller’s organ and belong to a multigene family. Their protein structures appear to have comparable ligand-binding cavities [52,67,69]. Our amino acid and multiple sequence analysis of these candidate molecules also revealed their shared binding protein properties: being small (average, 118aa), secreted proteins with predicted signal peptides in their N-terminals, and conserved cysteine residues are likely to form a strong disulfide bond, which enable them to stay stable in the extracellular environment (see Figure 1 and Figure 2).

These two candidates (NPC2 and ML), which were biasedly expressed in Haller’s organ, and have potential to be involved in tick olfaction, appear to comply with the binding protein standards proposed by Pelosi et al. [64]. The four criteria are: (1) a sufficient number of genes expressed in the same species (at least 12); (2) similarity to insect binding proteins (small, secreted, soluble); (3) having a ligand-binding pocket structure; and (4) stability. The authors used these criteria to identify potential candidates that might perform the role of semi-chemical binding proteins in non-insect arthropods, in which the authors primarily proposed NPC2s from *I. scapularis*. Therefore, we suggest that these candidate NPC2s and ML proteins may perform similar odorant-binding and transporting roles in ticks as the OBPs and CSPs perform in insects. However, the olfactory role of these individual candidates needs to be further confirmed with pertinent functional experiments.

#### Multiple Sequence Alignment on Representative Candidate NPC2s and MLs in Tick

Multiple sequence alignment analysis was conducted using amino acid sequences of candidate binding proteins involved in tick olfaction. Candidate molecules that are relatively highly expressed and biased to the fore-tarsus of ticks were selected as representative of the candidate NPC2 and ML proteins in our analysis. N-terminal signal sequences were predicted using the SignalIP5.0 online server https://services.healthtech.dtu.dk/service.php?SignalP-5.0 (accessed on 12 September 2022) and deleted before the alignment procedure. The analysis was carried out to highlight the shared properties of insect-binding proteins, including protein size, signal peptides, and cysteine residues.

Niemann–Pick protein type C2 (NPC2): The analysis revealed that all of the amino acid sequences of the small (average; 150 amino acids (aa)) NPC2 proteins were anticipated to contain a common N-terminal signaling sequence (average; 20 aa). The sequence alignment revealed that all of the candidate NPC2s commonly shared six conserved cysteine residues in their mature proteins (Figure 1).

Microplusin-like (ML): The candidate ML protein sequences revealed small proteins ranging from 99 to 150 amino acids (aa). ML proteins reported from *I. scapularis* have relatively longer sequences than those of *A. americanum.* Their mature proteins are predicted to have a signaling sequences in their N-terminals (average, 20 aa). All aligned candidate ML proteins appear to share four of their conserved cysteines, with the antimicrobial peptide microplusin, and three of the four cysteines with the previously reported candidate OBPLs (Figure 2). Members of these candidate ML proteins with six cysteines have also been reported by Josek et al. [52]. Like candidate NPC2s, ML proteins also appear to comprise a multigene family encoding small, secreted proteins that are potentially stable.

## 5. Miscellaneous: Poorly Evident Molecules Expressed in Haller’s Organ

Lipocalins are a superfamily of vertebrate odorant-binding proteins. Lipocalin resembles transcripts that are exclusively and/or differentially expressed in tick Haller’s organ. They appear to have signal sequences and share four conserved cysteines [52,67]. Furthermore, a proteomic study revealed allergen Der p7 and Der f7 proteins that were differentially expressed in the fore tarsus of *A. americanum* [64]. Allergens have a domain (START) that is usually found in highly stable proteins involved in olfaction [16]. The proteomic data have also revealed a neto-like protein previously linked to ionotropic glutamate receptor clustering and selectively expressed in the fore tarsi [18,51]. This protein may have a role in the dendritic development of the IR olfactory receptor architecture, according to Renthal et al. [67]. However, these molecules, including lipocalins, were limited in number with a low level of expression in the Haller’s organ, which poorly evidence their potential function as chemosensory-related candidate proteins in ticks. In spite of that, they can be considered as target proteins for future tick chemosensory-related relevant studies.

Odorant-degrading enzymes (ODEs): Several Haller’s organ-specific transcripts of ODEs, such as cytochrome P450, glutathione S-transferase, and superoxide dismutase, have been reported from *D. variabilis* [16], but not from that of *I. scapularis* [52]. Cytochrome P450 have been reported to be involved in the metabolism of odor ligands in insects [71], and to be inhibited by the common tick-repellent N, N-Diethyl-m-toluamide (DEET) in *D. variabilis* [72]. ODEs should be considered in future studies of tick control strategies, according to Carr et al. [16]. Despite the preferential expression of ODEs in Haller’s organ of *D. variablis*, although at a very low level, there is no sufficient information that indicates their potential chemosensory-related role in ticks. Comprehensive data are required, and future relevant studies should target ODEs. Members of the gene families that have been identified to be preferentially expressed in the forelegs of ticks (constitutes Haller’s organ) are summarized in Table 1, which have been proposed as chemosensory-related candidate molecules, and as having potential to be involved in tick olfaction.

**Table 1:** List of chemosensory-related candidate molecules preferentially expressed in forelegs, which ascribes Haller’s organ in four medically important tick species: *Amblyomma americanum* (Aa), *Dermacentor variabilis* (Dv), *Ixodes ricinus* (Ir), and *Ixodes scapularis* (Is).

Gene Family	Tick Specie	No. of Genes	FL Biased		In Palps	Descriptions Authors	
			EE	DE			
Chemoreceptors							
IR	Is	125	1	8	-	Primarily identified foreleg-biased IRs and GRs	[52]
GR	Is	28	-	6	-		
GPCR	Dv	-	8		-	Argued to biasedly express in Haller’s organ of *I. scapularis*.	[16]
**Potential binding proteins**							
NPC2	Aa	2	-	1	1	Low level in FL, and unquantifiable in palp	[67]
	Is	12	2	5	-	Expressed at various levels, with seldom FL biased	[52]
	Ir	2	-	1	1	Detected by IHC, good ligand-binding affinity	[69]
ML	Aa	4	2	1	1	Lack their antimicrobial basics, obtain a ligand-binding cavity,	[67]
ML (A-H)	Is	57	1	50	1	Shared common structure with OBPLs. (DE, RNAseq)	[52]
OBPL	Aa	2	-	2	1	Structure similar to insect OBPs, comparable cavity.	[67]
**Miscellaneous: poorly evident molecules expressed in** **Haller’s organ**							
Lipocalin	Aa	1	1	-	-	Have 2 to 5 conserved cysteine residues, and are expressed at various levels.	[67]
	Is	3		3	-		[52]
DMAL	Aa	2	2	-	-	Homolog model: insect Derp7 allergen proteins	[67]
Neto-like	Aa	1	1	-		Homolog model: insect neuropilin-1 proteins	[67]
ODEs	Dv	7	7	-	-	The first report on tick olfaction-related enzyme	[16]

FL, Foreleg; EE, exclusively expressed; DE, differentially expressed; IHC, immunohistochemistry.

Almost all of the relevant studies have focused on a limited number of tick species. The findings were mainly driven by the transcriptomes and proteomes of tick forelegs, which not only revealed a variable number, but also relatively fewer candidate proteins than similar studies on insects. This might be related to technique, the target species, or the nature of the tick chemosensory organ. Despite the limited number of relevant publications, our review has critically analyzed and discussed the available data and enabled us to determine the limitations of the existing studies and research gaps.

## 6. Evolutionary Relationship of Tick Chemosensory-Related Candidate Molecules

Phylogenetic analysis of tick olfactory-related candidate chemoreceptors, particularly members of the IRs and GRs from *I. scapularis*, has revealed a similar evolution to that of their counterparts in mites, while those of ticks appeared relatively more recently. Additionally, they have also been shown to be divergent from those of insects, according to Josek et al. [52]. Therefore, the authors suggested that ticks might have recently evolved adaptations to their novel chemical ecosystem. Hence, this phylogenetic evidence shows consistency with the previous reports on acarine chemosensory structures, in that tick and mite chemosensory sensilla have evolved from an ancestral pre-sensillum [14].

Despite the fact that a relatively small number of chemosensory-related potential binding proteins have been detected in ticks to date, the existing tick odorant binding protein-like candidates and their relatives from spider have been suggested as distantly related to that of insect odorant binding proteins [66]. In addition, phylogenetic analysis of olfactory-related putative-binding proteins (NPC2s) from some spiders (*Dysdera sylvatica* and *Pardosa pseudoannulata*) [66,73] has revealed that they share the same clade as those differentially expressed in tick’s Haller’s organ [67]. Furthermore, while most of the chemosensory-related microplusin-like (ML) molecules (MLA, B, and C family) from *I scapularis* have been shown to be specific to Ixodes, some appear to be related to those of mites (metaseiulus) and horseshoe crabs (limulus). However, their short coding sequence, along with that of rapidly evolving N-terminals, has resulted in a lack of sequence similarity with characterized proteins. This presents difficulty in evaluating the evolutionary relationship of these candidate proteins [52]. Therefore, based on these phylogenetic evidences, it can be suggested that the molecular basis of olfactory chemoreception in ticks might have a more recent common ancestor with their relatives in mites and spiders (possibly arachnids), compared with other arthropods.

## 7. Does the Molecular Basis of Olfactory Chemoreception in Ticks Differ from Insects?

Chemoreception in arthropods is determined by sets of large and small gene families [57]. In insects, volatile chemical signals are recognized by the sense of smell via the olfactory organs (antennae and maxillary palps), which ascribe olfactory receptors that detect odor molecules entering sensilla pores [74], in which odorant receptors (ORs) are responsible for a significant portion of their various olfactory skills [75,76,77]. However, ticks appear to have a unique olfactory organ (Haller’s organ) located in their foreleg tarsi [16], and no single OR has been detected to date. Instead, members of the ionotropic receptor (IR) family, which have relatively short and tick-specific protein sequences, with poor insect orthologues [52], have been suggested to be involved in tick olfaction.

Chemoreception in insects uses soluble proteins called odorant-binding proteins (OBPs) and chemosensory proteins (CSPs). They bind and transport volatile odorants to olfactory receptor neurons in the lymph surrounding the sensilla [64,78]. However, insect antennae have fewer binding protein genes than olfactory receptor genes [79]. None of the available tick genomes have detected OBP, while only one CSP [80] has been detected from *I. scapularis*. CSPs are multifunction proteins [68], which have been suggested to play a non-chemosensory role in ticks [64]. Instead, several transcriptomes and proteomic studies (Table 1), including a recent large-scale genome study [2], have identified multiple candidates belonging to a new class of binding protein family, including Niemann–Pick type C2 (NPC2) and the microplusin-like (ML). Evidence of two insect OBP-like genes has also been reported from *A. americanum* [67], but showed low amino acid sequence similarity to insect OBPs and was not part of the multigene family. Thus, it is possible that ticks have evolved new candidate binding proteins for odorant transport, unlike insects. This might indicate divergence on a molecular basis of tick olfactory chemoreception, compared with insects. This shows consistency with the presence of a unique tick chemosensory structure (Haller’s organ), and different number and type of chemosensory sensillum in ticks [16], unlike insects.

## 8. Major Limitations in Identifying the Tick Chemosensory-Related Candidate Molecules

Despite tick chemoreception being a topic of very recent research interest, several candidate chemoreceptors and a new class of binding proteins have now been identified as being involved in tick olfaction (Table 1). However, the significance of GPCRs (nematodes and vertebrate-like) [16,63], as well as insect-like OBPLs [67] and CSP [80], is unclear and is unsupported by strong data in ticks. Nevertheless, these cases, along with the yet unknown tick olfactory pathway, make it difficult to predict the key molecules involved, and this suggests that additional research is needed. Future studies should also focus on functional experiments, such as immunohistochemistry, ligand-binding assays, and RNA silencing in concert with tick behavioral assays, to determine the roles of individual candidate molecules in tick olfactory chemoreception.

Studies that have focused on identifying tick olfactory-related candidate molecules have largely relied on the tick paired-foreleg (bearing Haller’s organ) transcriptome and proteome investigations (Table 1). Haller’s organ is small and fibrous, making it difficult to target [16]. However, these studies have detected relatively smaller and varied numbers of target molecules, (Table 1) compared with similar studies on insects [81,82]. In addition, information on the distributions and molecular bases of the sensory neurons connecting the Haller’s organ to synganglion is limited, while it appeared to share similarity with those of mites, spiders, and some insects [19,28]. Flexible and innovative alternative techniques may detect additional target molecules. Relevant studies examining the insect antennae for a similar purpose may also provide important information regarding methodologies and techniques. Considering the wide expression profile of potential binding proteins in ticks [52,69], relevant studies need to also target beyond the Haller’s organ, to which comparative analysis following exposure of full ticks to odorants may contribute.

Because olfaction is a relatively recent topic of interest in ticks, few relevant research publications are available to compare with insect research. Most research on tick olfactory-related chemosensory molecules has been conducted on a small number of tick species (Table 1). The lack of abundant pertinent studies might be due to that fact that the first tick genome (*I. scapularis*) was reported in 2016 [18]. As different tick species exhibit genetic diversity (Jia et al., 2020), so variable outcomes of the existing relevant studies indicate the need for more comprehensive studies in the field. The recently sequenced large-scale tick genomes [2] have revealed several putative chemosensory-related molecules from six ixodid tick species. This should also increase the availability of molecular genetic references for different tick species.

Despite the limits observed in identifying the potential chemosensory-molecules potentially involved in tick olfaction, progress is being made. Existing Haller’s organ structure and functional studies using scanning electro-microscope and electrophysiological response investigations based on olfactory sensillum in Haller’s organ have contributed relevant knowledge and are promising techniques. Although chemoreception in ticks is a new research topic, the aforementioned study approach, methodology, and related shortcomings should lead to a greater understanding of how ticks perceive the chemical components of their environment. It may also contribute to identifying the key molecules mediating olfactory chemoreception in ticks.

## 9. Conclusions

Olfaction in ticks is mainly attributed to the unique sensory structure, namely the Haller’s organ. Studies using a scanning electron microscope and electrophysiological recordings have provided important information regarding the Haller’s organ morphology, its function, and its neurophysiological basics, despite the molecular chemoreception in ticks being a recent topic of research interest. The existing tick foreleg (contains Haller’s organ)-based transcriptome and proteome studies have revealed several candidate chemoreceptors and potential binding proteins. Bearing the absence of odorant receptors and only few gustatory receptors, olfactory reception in ticks presumed to heavily rely on ionotropic receptors, while niemann-pick type C2 and micoplusin-like proteins were proposed to represent a new class of binding proteins, unlike those of insects. These tick chemosensory-related candidate molecules appeared to evolutionary relate to those of mite and spider (possibly arachnids), however, independent functional experiments need to confirm their olfactory role in ticks. In addition to the largely unknown tick olfactory pathway, contradictions regarding candidate GPCR cascades and scant data supporting the potential role of insect-like odorant binding proteins and relevant neuropeptides in ticks has shown the extent of research gaps in the field. More comprehensive research will be required for underlying the molecular basis of olfactory chemosensation in ticks. We believe that this review, a recent topic of research interest in tick chemoreception, may contribute to attracting relevant researchers, and highlighting the knowledge gaps in the field. 

## Data Availability

All relevant data are within the paper.
